# Multi-feature fusion framework for sarcasm identification on twitter data: A machine learning based approach

**DOI:** 10.1371/journal.pone.0252918

**Published:** 2021-06-10

**Authors:** Christopher Ifeanyi Eke, Azah Anir Norman, Liyana Shuib

**Affiliations:** 1 Faculty of Computer Science and Information Technology, Department of Information Systems, University of Malaya, Kuala Lumpur, Malaysia; 2 Faculty of Computing, Department of Computer Science, Federal University of Lafia, Lafia, Nasarawa State, Nigeria; AIR University Islamabad, Kamra Campus, PAKISTAN

## Abstract

Sarcasm is the main reason behind the faulty classification of tweets. It brings a challenge in natural language processing (NLP) as it hampers the method of finding people’s actual sentiment. Various feature engineering techniques are being investigated for the automatic detection of sarcasm. However, most related techniques have always concentrated only on the content-based features in sarcastic expression, leaving the contextual information in isolation. This leads to a loss of the semantics of words in the sarcastic expression. Another drawback is the sparsity of the training data. Due to the word limit of microblog, the feature vector’s values for each sample constructed by BoW produces null features. To address the above-named problems, a Multi-feature Fusion Framework is proposed using two classification stages. The first stage classification is constructed with the lexical feature only, extracted using the BoW technique, and trained using five standard classifiers, including SVM, DT, KNN, LR, and RF, to predict the sarcastic tendency. In stage two, the constructed lexical sarcastic tendency feature is fused with eight other proposed features for modelling a context to obtain a final prediction. The effectiveness of the developed framework is tested with various experimental analysis to obtain classifiers’ performance. The evaluation shows that our constructed classification models based on the developed novel feature fusion obtained results with a precision of 0.947 using a Random Forest classifier. Finally, the obtained results were compared with the results of three baseline approaches. The comparison outcome shows the significance of the proposed framework.

## 1. Introduction

The advancement in computer technology and the World-Wide Web (WWW) has brought about growth in social media platforms such as Facebook, Instagram, Myspace, and Twitter to get connected with friends and families [1]. Hence, a large volume of social media data produced day after day requires analysis. People on social media share and publish messages, thereby making their personal information globally available. Identifying people’s subjective information, like people’s opinions, emotions, and sentiments, is made possible by such information. Analysis of people’s sentiment is a process of recognizing subjective information in source documents. The process of identifying people’s opinions (sentiments) about products, politics, services, or individuals brings a lot of benefits to the organizations [2,3]. Thus, It has become an important step in analyzing people’s sentiment [4,5]. The possibility of identifying subjective information is essential as it helps to generate structured knowledge that serves as a piece of important knowledge for decision support systems and individual decision-making [6]. Most of the social content found on the Web consists of figurative words such as sarcasm and irony. For example, the Internet Argumentation Corpus obtained from *4forums*.*com* consists of 12% sarcastic utterances [7]. Sarcasm is *“the use of remarks that mean the opposite of what one says*, *made to hurt someone’s feelings or to criticize something in a humorous way”* [8]. Sarcastic utterance represents a conflict between an individual’s motive for making the utterance and the actual composition. For instance, the sarcastic expression “I love to work on holidays!” shows a conflict between the clear statement “on holidays” and the articulation “love”. The contradiction and the sentiment polarities shift, proves that sarcasm is a unique form of sentiment analysis. Sarcasm is extremely contextual and topic reliant, and as a result, some contextual clues and shifts in polarity sentiment can assist in sarcasm identification in a text by determining the obscurity of the meaning and improving the overall sentiment classification of a large volume of user’s textual data obtained from social media. The insufficient knowledge of the situation “Context”, the environment, and the specific topic will result in difficulty detecting sarcastic utterance [9]. Context understanding is one of the main challenging phases of moderation content. The term “*Context*,” in sentiment analysis refers to supplementary support that may either increase or change the content polarity. However, the predictive performance of the sentiment classification will rely on context vector and learning algorithms to guarantee the reliability of the overall classification.

Sarcasm detection is the task of using NLP techniques to classify text with the properties and attributes of sarcasm. Automatic sarcasm identification is one of the major issues found in NLP because it is hard for humans to correctly classify utterances as sarcastic or non-sarcastic [10]. Also, there is a lack of correctly labelled sarcastic data employed to train classifiers [10,11].

Previous studies have attempted to identify sarcasm in a tweet using different feature engineering approaches. Literature on sarcasm detection reveals that these existing methods suffer two main problems [12–15]. One, the context of the words in representation is ignored [16]. Consequently, different expressions can possess similar vector representation. Two, data sparsity in vector representation since each expression has a word limit. This issue can create a problem during the model training because some words could be seen in the testing set only but never found in the training set, making most of the training feature vector sparse.

The utilization of contextual features in sarcasm identification has recently gained ground on social media platforms. Microblog data contains highly contextual information. As a result, the application of content-based linguistic features in sentiment classification becomes relatively ineffective and requires the addition of some contextual clues [17]. A study conducted by Wallace, Kertz [18] investigated this fact by indicating the failure of traditional classifiers in a situation wherein human requires additional context. In general, the most related techniques for sarcasm identification produces low performance and results not generalized when there is a sparsity of data and reliance on only content features for prediction. However, addressing these issues requires additional information, such as contextual information. For successful operation in sentiment analysis settings, the sarcasm identification system is required not only to identify sarcasm. Still, it should also perform the additional complex task of differentiating it from negative and positive sentiment tweets. In doing so, most related techniques have always focused on the content-based features [11] that consider tweets’ contents only. However, most studies in linguistic concepts related to sarcasm maintain that employing contextual features that consider tweet context enhances predictive performance [19].

Previous studies have proposed various feature engineerings methods such as the N-gram, Bag-of-words and word embedding for sarcasm identification in social media [14,20,21]. Even though few studies have implemented conventional text classification-based feature engineering methods for sarcasm detection, literature studies [12–15] reveals that most current methods face various issues that need to be resolved to improve the sarcasm identification framework. This includes one, the context of the words is ignored in representation in the sentence since it is only concerned with the occurrence of the word. This leads to ***loss of contextual information*** and in turn, the semantics /meaning of words in the expression [15,22]. Two, ***Sparsity of training data issue;*** due to the word limit of microblog, the value of feature vector for each sample constructed by BoW produces null feature, making the modeling data sparse [13,23].

Therefore, it is important to explore more approaches to overcome these drawbacks and enhance predictive performance in sarcasm classification. Even though the current technique may have produced promising results in some case for Twitter data with 140-word character tweet, as Twitter has extended the word usage from 140 to 280, this approach is no longer effective for Twitter data. Thus, there is room for improvement for larger databases [24]. Hence, there is a need to carry out this research.

Applying a machine learning algorithm may offer successful or unsuccessful sarcasm detection results because constructing a successful classification model relies on various factors. The key factor is the feature utilized and the independent features in the learning algorithm that associate easily with the class instance. Effective feature selection from both positive and negative classes requires a substantial effort in constructing the classification model. Accordingly, this study objective is to develop a Multi-feature Fusion Framework for sarcasm identification to address the context of words and training data sparsity issues in sarcasm expression. The key contributions of this study are provided below:

- The study proposes and extracts various sets of comprehensive features that consist of lexical, length of microblog, hashtag, discourse markers, emoticon, syntactic, pragmatic, semantic (GloVe embedding), and sentiment related features which are selected based on observations from the characteristics of the data and evidence from the literature. The observation has been transferred to suitable features, which are now experimented with to enhance the classifiers’ performance.
- A novel feature extraction algorithm for extracting discriminative features, and two stages classification algorithm, by considering the lexical feature in the first stage and fused features in the second stage for sarcasm identification. This is the first study on sarcasm identification that proposes feature extraction algorithm and two classification stages on conventional machine learning models to the best of our knowledge.
- We developed an effective Multi-feature Fusion Framework for sarcasm identification to overcome the limitations mentioned above in the most related techniques by addressing the context of words and data sparsity in expression for sarcasm classification.
- The study investigated the impact of feature selection methods to uncover the mixtures with the substantial discriminative ability that can produce an enhanced performance. Two feature selection algorithms were tested, namely, Pearson correlation and information gain, to reveal the most important proposed features for feature fusion. It also reduces feature dimensionality, lessens classification time, and eliminates redundant features insignificant to the performance results.
- The sarcasm detection model obtained a precision of 0.947 and an f-measure of 0.946 using a random forest classifier. The obtained results for the proposed Multi-feature Fusion Framework were compared with four baseline approaches, and the evaluation indicates the importance of the proposed framework for sarcasm analysis.
- The rest of the article is arranged thus: In section 2, a description of related work is provided. Section 3 described the materials and methods used in this study. In section 4, the experimental setting is presented. Section 5 provides the empirical results and discussion, whereas section 6 finally concludes the article. The lists of abbreviations and definitions utilized in this study are depicted in Table 1.

**Table 1:** Lists of abbreviations and definitions.

Abbreviations	Definitions
API	Application programming interface
ARTK	Automatic retrieval of tweets using keywords
BERT	Bidirectional Encoder Representation from Transformer
BoW	Bag-of-words
CI	Content information
CNN	Convolutional Neural network
CPU	Central processing unit
DT	decision tree
FN	False negative
FP	False positive
GB	Giga byte
GFI	Grammatical function information
GloVe	Global vector
KNN	K-nearest neighbour
LIWC	Linguistic inquiry and word count
LR	logistic regression
LSTM	Long short term memory
NLP	natural language processing
NLTK	Natural language toolkit
POS	parts of speech
RAM	random access memory
RF	Random forest
SVM	support vector machine
TAALES	Tools for automatic analysis of lexical sophisticated
TF-IDF	Term frequency-inverse document function
TN	True negative
TP	True positive
WEKA	Waikato Environment for Knowledge analysis
WWW	World wide web

## 2. Related work

This section provides the literature survey of the current study on automatic sarcasm identification focusing on feature engineering. The summary of the related work is depicted in Table 2. A detailed analysis of the feature engineering techniques employed in sarcasm classification tasks can be found in a systematic literature review conducted in [25].

**Table 2:** Summary of the related works on feature engineering aspect for sarcasm detection.

Study	Features employed	Limitation of the study
[10]	Pragmatic and lexical features	Difficult in the interpretation and understanding by a computer program as it is based on pragmatic feature
[17]	Emoticon, punctuation mark, positive interjection, and quotation mark	The study focused only on deeper linguistic analysis and fail to consider word context
[26]	Sentiment related, lexical, punctuation related and pattern related features	Due to the smaller amount of the training set, the extraction of all possible sarcastic patterns are not covered
[27]	POS tags, interjection related and punctuation related features	Relies solely on pragmatic factors, word semantics and context ignored
[28]	Hyperbolic features (interjection words and intensifiers)	It relies on the content feature only
[29]	Lexical density, number of the intensifier, first-person singular and plural, third-person pronoun.	Features extracted relies only on the earlier acquired tweets for prediction
[30]	Function and content word, bigram, and PoS	Unable to differentiate the various form of sarcasm; rather, the authors deemed it as a 2-class issue
[31]	LIWC, TAALES, bigram, semantic, Psycholinguistics, and statistical features	Unable to work with synthetic (POS) features, including the order to appearance.
[32]	Text, speech, and video features	An over-fitting problem in a complex neural model.

Bouazizi and Ohtsuki [26] investigated a pattern-based method for detecting sarcasm in tweets. The authors defined four categories of features; sentiment related, punctuation related, pattern related, and lexical features for detecting sarcastic expression in tweets. They proposed an effective and reliable pattern for sarcasm identification so that words are grouped into two separate classes: “CI” and “GFI”. While the CI emphasizes the importance of the word’s contents in the expression, the GFI class concentrates more on the grammatical function of the word. However, the Random Forest classifier was employed for prediction purposes and an accuracy of 83.1% with a precision of 91.1% was obtained. Still, the study relied more on the patterns of the words in the expression, which are not sufficient in capturing the sarcastic sentiment. Riloff, Qadir [33] presented an approach for detecting different kinds of sarcasm whereby a contradiction exists between positive sentiment and negative situations. They proposed a ‘bootstrapping algorithm’ that utilizes a single seed word that automatically identifies and learns a phrase that shows the contradiction between negative situations and positive sentiment in sarcastic tweets. The authors created two benchmark approaches and employed the LIBSVM library to model SVM classifiers in measuring the model’s performance. However, a precision of 64% and 39% recall were obtained by employing SVM on unigram and bigram features. Thus, this method performed optimally well but many positive classes are not captured in the classes mentioned above on sarcasm. Also, the method depends on the occurrence of every likely “Negative situation” on the training data. In such cases, it will lower the predictive performance when working with new tweet data.

In addition to pragmatic features to the lexical features, Muresan, Gonzalez-Ibanez [10] developed a technique that creates a dataset of sarcastic tweets, whereby the tweet owner determines if a tweet is sarcastic or not. They experimented with the impact of lexical and pragmatic features on the predictive performance during their study to identify sarcasm in tweets and rated features based on their classification contribution. The empirical analysis indicated that incorporating pragmatic features on lexical features increases the predictive performance on sarcasm detection on Twitter. Consequently, this study relied only on the content features without considering the contextual information, improving the predictive performance in sarcasm classification. In another study, Carvalho, Sarmento [17] experimented with the effect of surface pattern that consists of the emoticon, punctuation mark, positive interjection, and quotation mark for Irony detection in news articles. The empirical analysis improved the accuracy of the predictive performance from 45% to 85% with the surface patterns compared with the deeper linguistic analysis that produced low accuracy, which is a limitation of this study. In a related study, Bharti, Naidu [28] examined hyperbolic-based features for sarcasm identification on Twitter data. The study considered interjection words and intensifier (an adverb or an adjective) in a sentence as hyperbole content. To extract those features, an algorithm was developed for the extraction of n-gram of the hyperbolic phrase feature for classification. The experimental analysis indicated that the use of interjection word and intensifier enhances classifiers performance on sarcasm classification. However, the study relied only on the hyperbolic features only for sarcasm classification, which is insufficient to capture all the sarcasm sentiment in a given tweet. Also, the feature extraction algorithm developed follows a specified pattern, which is not the best practice for the feature extraction process. Rajadesingan, Zafarani [29] went deeper and looked into the psychology involved in the sarcastic expression. Their study presented behavioural modeling for sarcasm detection by identifying various forms of sarcasm and their existence on Twitter data. The study showed the significance of historical information acquired from the past tweet for sarcasm identification. Though the approach looks very effective in such an instance, however, it cannot perform well in a situation whereby the past knowledge about the user is not known. This is because most of the features employed for classification were extracted from the dataset based on the user’s past tweets for the decision making. Thus, it is difficult to apply the approach in real-time tweets streaming whereby users are randomly publishing tweets due to the fast growth in the knowledge base, which requires the repetition of training on data each time new tweet data is acquired. In another study, ONAN, TOÇOĞLU [34] proposed a Turkish news article’s satire detection method. The authors employed linguistic Inquiry and word count software for feature extraction by considering the linguistic and psychology feature sets. In this study, ensemble learning, five deep learning architecture, and word embeddings scheme were considered. The experimental analysis of the proposed approach showed that the deep learning approach outperformed other approaches, which showed the significance of the proposed methods. However, the deep learning approach employed in this study uses a word embedding learning algorithm as a standard approach for feature vector representation, which ignores the sentiment polarity of the words in the sarcastic expression

With the hybrid of supervised and unsupervised learning, Mukherjee and Bala [30] presented a method that provides knowledge to the system for interpreting the author’s linguistic style that considers different sets of features for sarcasm identification in microblogs. They used authorial style-based features in their study using Naïve Bayes and fuzzy clustering in the classification phase. The experimental analysis indicates that supervised and unsupervised learning with the inclusion of features that are independent of text produced better accuracy in sarcasm detection. However, the approach is only limited to authorial style-based features. In conjunction with the machine learning approach, current researchers in this domain have shifted their attention to the concept of deep learning. Ghosh and Veale [35] proposed a deep neural model for sarcastic analysis in tweets. The author integrated machine learning and neural network models that use CNN, LSTM, and distributed neural networks. However, the empirical analysis indicated a better performance by producing an F-score of 92%, compared with the benchmark approach. Recently, the application of multi-tasks learning has gained recognition and has been demonstrated in various NLP tasks, including key phrase boundary detection [36] and implicit discourse relationship detection [37]. In a related study, Majumder, Poria [38] proposed a ‘multitask learning framework using DNN’ for sentiment and sarcasm identification study. In their research, they demonstrated that the two tasks are related and, as a result, modelled the two tasks using a single neural network. The experimental results slightly performed better than the existing approach, revealing that the multitask network improves sarcasm classification and polarity shift in the sentence. However, contextual information was not considered in this study. In another study, Onan and Toçoğlu [39] presented an effective sarcasm identification framework on social media data by considering a deep learning approach with neural language models such as FastText, GloVe, and word2vec. The authors introduced inverse gravity moment based on weighted word embedding with trigram. The empirical analysis of the proposed framework attained an accuracy of 95.3%, which indicates the effectiveness of the proposed framework. However, this study uses a word embedding learning algorithm as a standard approach for feature vector representation, which ignores the sentiment polarity of the words in the sarcastic expression

In a recent study, [40] proposed the word sense disambiguation deep learning method. In the proposed approach, the sense path in a target context is modeled by exploiting the domain-specific background knowledge from WordNet by employing the word embedding feature extracted from an external corpus. The method revealed the hidden semantic relationship within word sense by utilizing the ‘PageRank algorithm’ to exploit sense path via WordNet structure while representing the text context target with latent semantic analysis. However, the study employed only word embedding features for sarcasm classification, which has a limitation in capturing the sentiment polarity of the sentence in a given utterance. Thus, the word embedding feature is not enough to capture all the sarcasm sentiment.

In a related study, Duan, Luo [41] proposed a new semi-supervised learning method that considers both training and testing sets for sentiment classification of stock text messages. The method was proposed to resolve the issue common in short message modeling, such as data sparsity in mathematical representation. Moreover, the author constructed a Generative Emotion Model with categorized words (GEM-CW) to extract sentiment features from both training and testing sets. However, the extracted features were more discriminative for sentiment classification than those derived using the conventional approach that only considers training sets. The analysis results indicate that the proposed learning approach and the model are significant for sentiment classification in short text and can attain better results than the traditional methods. Thus, the approach may not perform well in a long text document. In a recent study, Eke, Norman [42] proposed a context-based feature technique for sarcasm identification. The author tested the predictive performance of the deep learning and BERT model on the benchmark datasets. The authors further proposed a feature fusion technique that consists of BERT feature, GloVe embedding features, sentiment related features, and syntactic feature, and compare the results with the two learning models. However, the feature fusion approach outperformed both the deep learning and BERT model, which shows the significant of the feature fusion approach.

The literature review above shows that most of these machine learning models for sarcasm classification have always focused on expression contents only, without paying more attention to contextual information and failing to capture the semantics and co-occurrence statistics in sarcastic expression. Thus, this study seeks to address the gap.

## 3. Materials and methods

### 3.1 Data collection

The sarcasm identification process begins with the acquisition of a suitable dataset. Dataset is very crucial in any data mining studies. The Automatic Retrieval of Tweets using the Keywords (ARTK) approach has been employed in this study to acquire the dataset for sarcasm classification purposes. The Dataset acquisition was carried out by using the Twitter streaming API for both sarcastic and non-sarcastic collection. Data collection for this research took place between June 2019 and September 2019. Twitter is a leading microblog site that enables users to exchange their ideas, news, and emotion with their co-users. One of the major advantages of Twitter data is that one can collect as many tweets as possible because people post messages daily. With the Twitter application program interface (API), a connection between Twitter servers and users is provided to make archived tweets easily accessible. API facilitated the extraction of public tweets. Each of the tweets extracted using the API provides extensive information about the users. [43], which includes the user identification, URL, user name, user account information, tweet text (which is the major textual data required for the analysis as it contains the emotional, behavioural, and other information and thoughts) [44]. This information has been utilized to construct a feature set for the effective classification of Twitter data [44,45]. It has also been used to develop a proposed multi-feature fusion framework by identifying the machine learning model’s significant features for model training in differentiating between the sarcastic and non-sarcastic expression.

To build the datasets of sarcastic and non-sarcastic, self-annotated tweets by tweets owners were streamed from Twitter and utilized. The scope of this study covered only tweets composed in the English language. Tweets expression having the hashtag ‘#sarcasm’ or ‘#sarcastic’ is considered sarcastic, a similar concept used in [30,46]. However, tweets without such hashtags are considered non-sarcastic by following the same concept utilized [47] or tweets with keywords #notsarcasm or #notsarcastic [30]. In this research, balanced tweet datasets of 29,931volume of tweets that contained 15,000 sarcastic and 14,931 non-sarcastic tweets are used for the analysis. The datasets consist of real-time tweets covering the aspects of politics, education, technology, etc. The summary of the dataset is depicted in Table 3.

**Table 3:** The summary of dataset description.

Data source	Twitter
Data collection approach	Automatic retrieval of tweets using keywords (ARTK)
Language of tweets	English
Data classes	Sarcastic and non-sarcastic
Search period	Between June 2019 and September 2019
Sarcastic data volume	15,000
Non-sarcastic data volume	14,913
Total volume of data	29,931
Annotation	Self-annotated by tweet owner
Sarcastic annotation	‘#sarcasm’ or ‘#sarcastic’
Non-sarcastic annotation	#notsarcasm or #notsarcastic or without any hashtag

### 3.2 Data pre-processing

One of the drawbacks of obtaining data set from Twitter is the noise that comes along with the data. Twitter data (tweets) may be in the form of simple text, user’s mentions (@user), and reference to URLs or a content tag, also known as hashtags (#). In this stage, the sarcastic and non-sarcastic data were pre-processed to prepare before the feature extraction and classification task. This is carried out in various steps to remove noise from the sarcastic datasets, including retweets, duplicates, numerals, tweets written in other languages, and tweets with the only URL. These noisy data do not contribute to the enhancement of classification accuracy and are, therefore, eliminated. The text data were converted to the lower case and other basic pre-processing techniques such as tokenization, stop word removal, spell check, stemming, and lemmatizing. POS tagging is also employed, implemented using the Python library and Natural Language Processing (NLP) toolkit. They are briefly described below.

***Tokenization***: This is a process of splitting words or sentences into smaller chunks called tokens such as words, phrases, and symbols that are useful on their own. The tokenization process also eliminates the empty white space characters found in textual documents. A token refers to a sequence of characters found in a particular document joined together to create an appropriate semantic unit useful later during the analysis. Thus, the tokenization output becomes an input for future analysis. Tokenization tasks can be performed using the NLP toolkit.

***Stop word removal***: These are common words that consist of articles and prepositions (such as a, an, the, etc.) that do not have an influence on the context of the expression and do not have any contribution to the text analysis. NLTK corpus stop word was employed to remove the stop word from the data set. It should be noted that empirical analysis was performed to examine the model performance in the existence or nonexistence of stop word in the text. The reason is that few studies on text classification indicated that the absence of stop words reduces the performance of the classification [48,49]. Contradictorily, several pieces of research demonstrated that the existence of stop words in the text reduces classification performance. In our study, the experimental analysis of the stop word indicated that stop words lower the performance results due to the noisy factor [48,50,51]. Therefore, stop words were eliminated, in turn, to improve the classification results. The pre-processing phase is taken as an input to the next classification phase, known as the feature engineering phase.

***Spell correction***: This is a process of checking for the text’s spelling to correct the wrong spelt text. A PyEnchant [52] spell checker python library was employed to correct all the misspelt words.

***Stemming*:** Stemming is the restoring of the derived words into its root form or obtaining the root word called the stem, by removing the prefixes and suffixes from the word. The stemming process reduces the keyword space’s number and enhances the classification performance when a single keyword is obtained from a different form of the keywords. For example, the word ‘stealing’ can be stemmed to ‘steal’. However, the Port stemmer library was employed for the word stemming task. Various studies stated that the stemming procedure contributes to the classification performance as found in [53,54]. Thus, the stemming process was performed in this study to enhance the classification performance.

***Lemmatizing***: The removal of prefixes and the suffixes in a derived word sometimes render the word meaningless. Lemmatization is another normalization technique that truncates the inflectional of a word using morphological and vocabulary analysis of a particular word to transform it into a dictionary form. Lemmatizer, therefore, inputs the missing characters to the stemmed word to bring meaningfulness out of it. This procedure normalizes the word into basic forms. Unlike stemming, lemmatization does not yield the word stem but substitute the suffix of the input word with a different word to generate its normalized form. For instance, the word ‘concluded’ can be stemmed to the word ‘conclud’, which can then be lemmatized to the ‘conclude’.

***Parts-of-speech (POS) tagging***: POS tagger reads the textual documents and allocates parts of speech to each token based on its definition. The tagger allocates various parts of speech such as verb, noun, adverb, adjectives, conjunctions, interjections, etc. Most computational sciences application needs fine-grained POS tagging. For instance, noun tagging can exist in different forms, such as singular nouns, possessive nouns, and plural nouns. POS tagger uses various notations. For example, NN notation represents a singular common noun, NNS represents plural common nouns and NP for singular proper noun. However, the POS tagger for tagging uses stochastic and rule-based algorithms.

For example, in a given sample tweet below.

“@realDonaldTrump @POTUS Mr Trump, I can vouch for this guy. Took him. Real salt of the earth type. Top bloke all round is our Stephen #Sarcasm https://t.co/G5N24J5nMX”

Data preparation for the sample tweet is provided below.

Initially, the tweet sample is broken-down into stream of tokens as illustrated below. The tokens are thus, converted to lower case for uniformity purpose.

[‘@realdonaldtrump’, ‘@potus’, ‘mr’, ‘trump’,’,’, ‘i’, ‘can’, ‘vouch’, ‘for’, ‘this’, ‘guy’,’.’, ‘took’, ‘him’,’.’, ‘real’, ‘salt’, ‘of’, ‘the’, ‘earth’, ‘type’,’.’, ‘top’, ‘bloke’, ‘all’, ‘round’, ‘is’, ‘our’, ‘stephen’, ‘#sarcasm’ ‘https://t.co/G5N24J5nMX’]

Next, URLs, user mentions and numbers in a given tweet sample is substituted using the placeholders as shown below.

[‘AT_USER’, ‘AT_USER’, ‘mr’, ‘trump’,’,’, ‘i’, ‘can’, ‘vouch’, ‘for’, ‘this’, ‘guy’,’.’, ‘took’, ‘him’,’.’, ‘real’, ‘salt’, ‘of’, ‘the’, ‘earth’, ‘type’,’.’, ‘top’, ‘bloke’, ‘all’, ‘round’, ‘is’, ‘our’, ‘stephen’, ‘URL’]

Lastly, text normalization such as stemming and lemmatization is performed to transform words into their root form, active tense, singular and present tense. This process allows easy parsing and effective feature extraction from the data.

[‘AT_USER’, ‘AT_USER’, ‘mr’, ‘trump’,’,’, ‘i’, ‘can’, ‘vouch’, ‘for’, ‘this’, ‘guy’,’.’, ‘take’, ‘him’,’.’, ‘real’, ‘salt’, ‘of’, ‘the’, ‘earth’, ‘type’,’.’, ‘top’, ‘bloke’, ‘all’, ‘round’, ‘is’, ‘our’, ‘stephen’, ‘URL’]

### 3.3 Proposed multi-feature framework for sarcasm identification

This section describes the multi-feature fusion framework for sarcasm identification. In this framework, the Automatic Retrieval of Tweets using a Keyword (ARTK) approach was employed to acquire dataset utilized in this study. The dataset undergoes the pre-processing stage, as explained in section 3.2. Nine sets of proposed features as presented in subsection 3.4 are extracted from the dataset. The output of the extracted features is employed to develop a Multi-Feature Fusion Framework using two classification stages. However, the decision to choose the best classifier for a particular dataset is quite challenging. In existing studies, two or more machine learning algorithms are tested to find the best algorithm since it is difficult to find a single classifier that can attain the best performance in all application domains [55]. This is because of the variations in the philosophy of the learning process. Thus, five different classifiers: DT, SVM, LR, K-NN, and RF, have been employed to assess the feature fusion framework’s model performance. As a guide in selecting classifiers utilized in this study, three points have been used to scale down the selection. One, specific literature on the classification algorithm for sarcasm detection helped in classifiers selection. The distinction of the machine learning model may be restricted to a particular domain [56]. Therefore, the literature review carried out in section 2 serves as a guide in selecting the classifier. Two, a text mining study review was also used as a guide for model selection [57,58]. Three, the comparative results on comprehensive datasets also guided in selecting the classification algorithm [59]. In this study, feature analysis and selection schemes were also investigated to identify and select the discriminative features and eliminate the redundant ones that do not contribute to the classification results.

**Fig 1:**
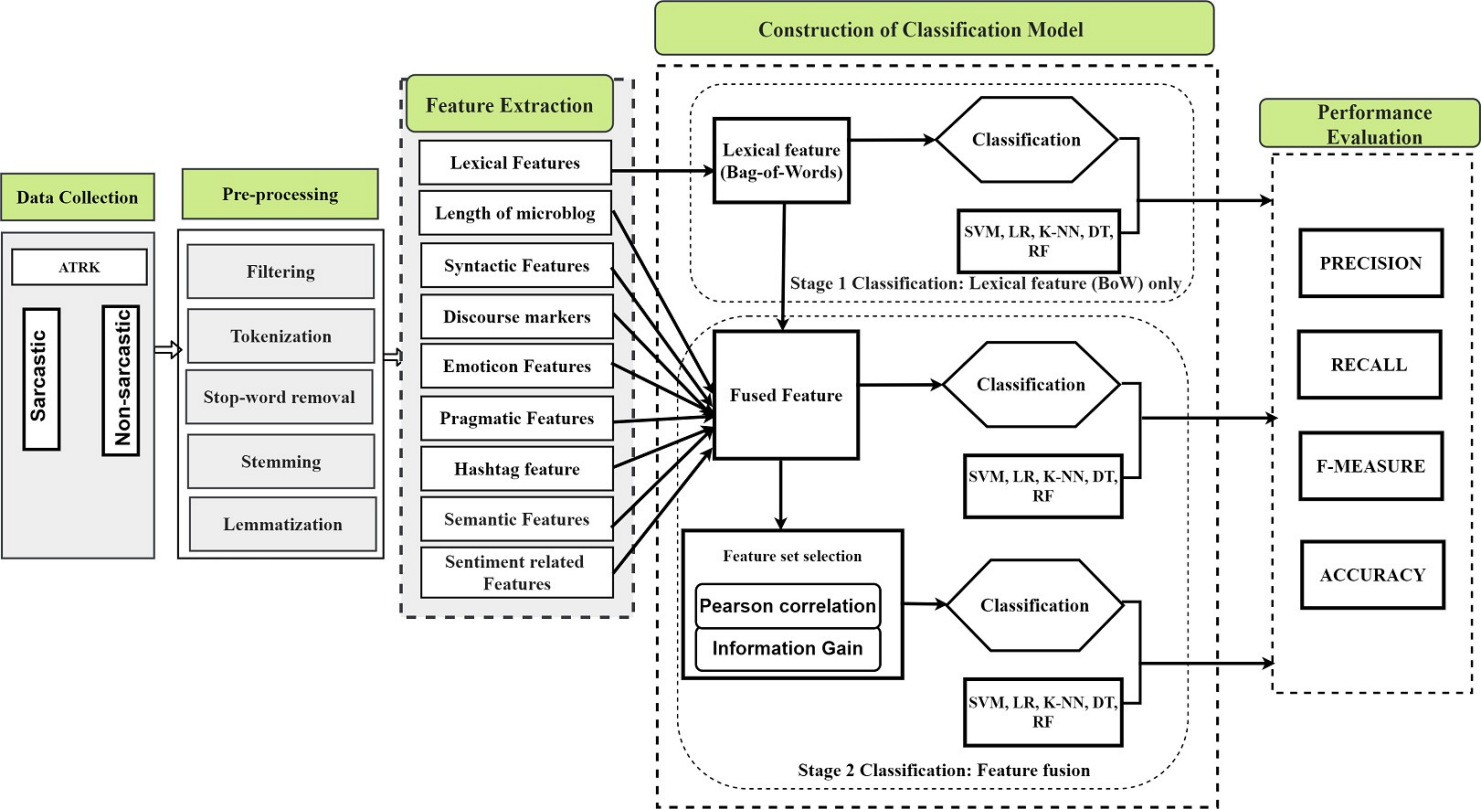
Multi-feature fusion framework for sarcasm identification.

**Fig 2:**
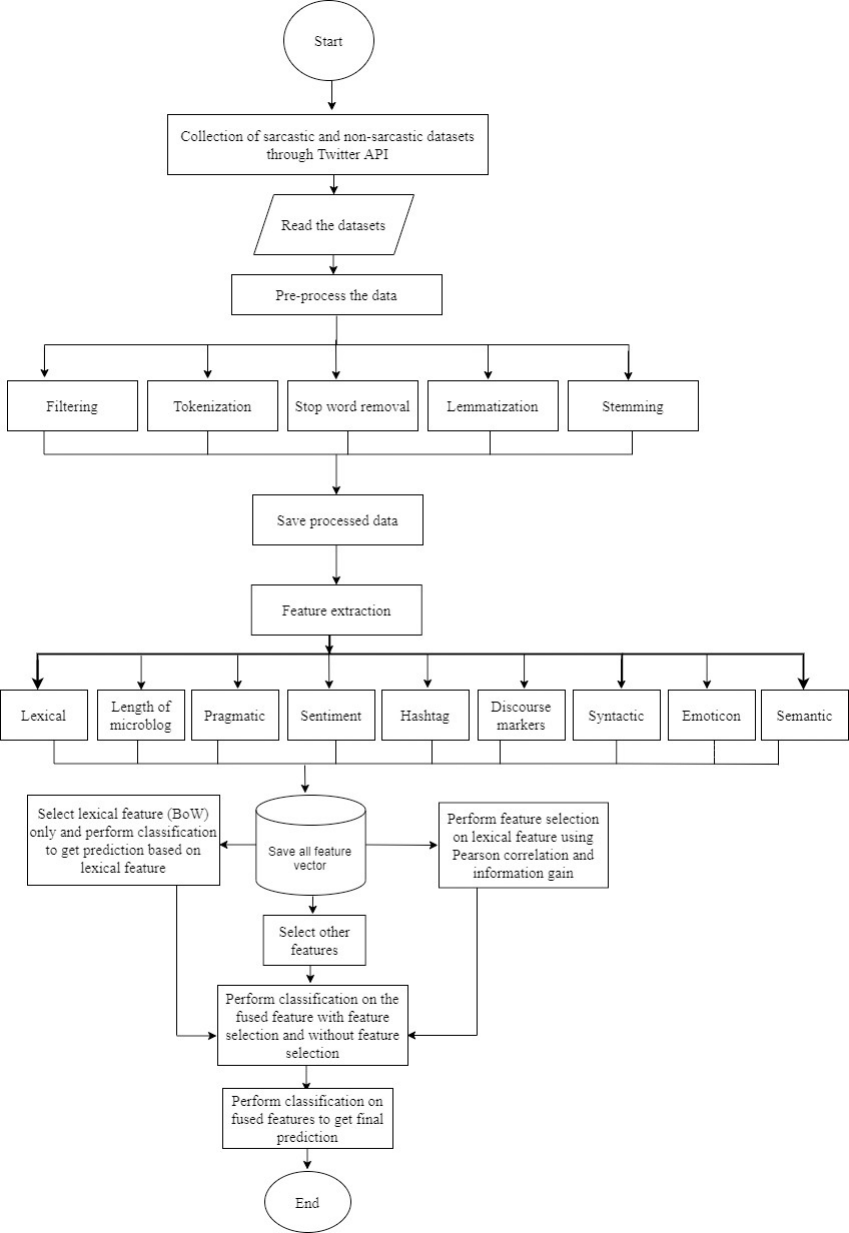
The flowchart of the proposed methodology.

Various experiments were performed to measure the efficiency of the proposed framework for sarcasm identification with the dataset. This study utilized ‘Precision’ as the major performance evaluation. However, other performance metrics such as recall, f-measure, and accuracy have been employed to supplement the framework evaluation. Lastly, the proposed feature fusion framework was used to compare three state-of-the-art baseline feature engineering technique studies on sarcasm identification. Thus, the evaluation aims to know how suitable and adequate the proposed framework is in identifying sarcasm and examine which approach is more appropriate in classifying text as sarcastic or non-sarcastic. The proposed framework is developed to overcome the aforementioned limitations of most related techniques by addressing the context of words and data sparsity in expression for sarcasm classification. The proposed framework is depicted in Fig 1 whereas the flowchart methodology is shown in Fig 2.

### 3.4 Feature engineering

Feature engineering is one of the key processes in any text classification task. In the feature engineering stage, the features that have discriminative power in differentiating sarcastic from the non-sarcastic text are extracted from the processed data. Apart from feature extraction, other feature engineering schemes such as feature representation and subset feature selection are investigated in this stage. Previous studies have relied on content-based feature, for example, Bag-of-words features, in isolation for sarcasm detection without considering contextual features. Performance results obtained with content features revealed that these features alone are not sufficient to accurately capture all the sarcastic tendency in the text. In order to enhance the performance of the model, some comprehensive novel features have been proposed to augment the content features. These features are presented in this section for the development of feature fusion. They include lexical, length of microblog, hashtag, discourse markers, emoticons, syntactic, pragmatic, semantic (GloVe embedding), and sentiment related features for sarcasm identification. These features were extracted from tweet content. These features, as described below, were employed in conjunction with the classification algorithm to construct a model for sarcasm identification. As stated in the previous section, we utilized SVM, RF, LR, KNN, and DT classifiers. Investigation on all proposed features was performed (see result section) by utilizing the feature selection technique to identify the discriminative features for fusion with promising results. The discussion on the proposed features is provided below, and the summary is depicted in Table 4.

**Table 4:** The summary of proposed features for sarcasm identification.

NO	Groups	Features
1	Lexical features	Features based on bag-of-words which uses TF-IDF as a lexical level feature
2	Length of microblog	Length of microblog feature
3	Hashtag feature	Positive hashtags, Negative hashtags, co-existence of positive and negative hashtags
4	Discourse marker features	Discourse markers such as temporal compression and counter factuality
5	Emoticon features	Positive, negative and sarcastic emoticon
6	Syntactic features	Laughing expression, POS (Noun, verb, adverb and adjectives), and Interjection.
7	Pragmatic features	Exclamation mark, Question mark, Ellipsis, Quoted word, All capitals, Repeated vowels.
8	Word embedding	GloVe embedding features
9	Sentiment related features	Positive sentiment words, Negative sentiment words, Highly emotional positive content, highly emotional negative content, contrast related features between the sentiment components

***Sentiment related feature*:** The most common form of sarcasm that occurs in social media is a whimper. In whimper, the composer of sarcastic utterance uses positive sentiment to describe a negative situation. In this regard, sarcasm’s expression uses contradicting sentiment that can be observed in the expression of a negative situation using the positive sentiment, as found in the study on sarcasm analysis conducted by Riloff, Qadir [33]. For example, ‘I love being cheated always.’ In this study, we investigated a contradiction between the word’s sentiment and other components in the tweets to recognize such sarcastic statements. To this end, sentiment related features are extracted from each tweet and counted. In this study, seven subsets of sentiment related features are defined, which include positive sentiment words, negative sentiment words, highly positive sentiment words, highly negative sentiment words, co-existence between positive sentiment & negative sentiment words, co-existence of positive and negative sentiment words with hashtags, and co-existence of positive and negative sentiment words with emoticons. To extract sentiment related features from the tweet’s content, a dictionary that consists of positive words and negative words is created using the SentiStrength [60] database. SentiStrength is a sentiment lexicon that utilizes linguistic rules and information to detect English text sentiment. The lexicon usually provides the polarity sentiment (positive and negative) of words like question, negation, emotion, booster, idioms, slangs, and emoticons. The sentiment score uses integers ranging from -5 to +5, in which the larger absolute value represents the stronger sentiment. The first two features are extracted using the two lists by computing the number of sentiment words that have a tendency of being positive or negative. The next two features (highly positive and negative positive words) are extracted by checking if any of the positive or negative sentiment words are associated with highly emotional parts of speech (adjective (JJ), verb (RB), adverb (VB)) tags tweets. If it occurs, an integer 1 is recorded otherwise; 0 is recorded. Lastly, the last three features are extracted by checking the co-existence of positive sentiment & negative sentiment words, positive sentiment & negative sentiment words with the hashtag, and positive sentiment & negative sentiment words with an emoticon in the same tweet by recording integer 1 if there is co-occurrence otherwise 0. Therefore, the sentiment-based feature contains seven subsets of feature.

***Pragmatic (punctuation related) features*:** In this study, we utilized punctuation marks as pragmatic features. Punctuation has an important effect on text analysis, especially in sentiment analysis. Punctuation symbols are mostly used as an explicit mark that brings out the sarcastic expression in the text. In punctuation related features, six different sets of features were considered and were extracted from tweets content. To extract punctuation marks from the tweets, a regular expression is employed to check the punctuation marks present in the sarcastic expressions. After that, the number of times each of them is used is computed. Firstly, the numbers of question marks were calculated and extracted as a feature (?). The second feature was obtained by counting the number of exclamation marks in the text (!). The third feature calculated the number of ellipses (.) in the text. The fourth feature considered capitalization in the text and computed the number of occurrences, i.e. it searches for the word that is “All-capitals” and extracted it as a feature in the text. The fifth feature calculated the quoted words, which are the words that are in a quote, and added it as a feature. Lastly, the sixth feature calculated the repeated vowels in the text and added it as a feature. Thus, these six features formed a feature set for related pragmatic features.

***Length of microblog feature***: A study conducted in [61] noted that that opinion mining results could be influenced by the number of words that a text expression contains. Also, the author reported that most of the non-sentimental statements commonly occur in a longer text. In such an instance, there is difficulty in analyzing such text to find accurate sentiment. Length of microblog defines the depth of sentence in conversation, and it is important to determine if speech is sarcastic or not. However, this feature was mentioned in sentiment analysis in [61]. The impact of deploying length of words for sarcasm identification is yet to be investigated by any studies. After careful analysis, I discovered how some of the sarcastic text differed in lengths based on utterance and proposed the features for sarcasm detection. Even though it has been preliminarily studied in sentiment analysis, this is the first study to comprehensively investigate and implement the inclusion of the features for distinguishing sarcastic and non-sarcastic tweet. Thence, the length of the text is considered as a feature for sarcasm identification in this study. To extract length of the microblog feature, each tweet’s length is calculated and measured as an integer in the text by employing a “Counter” python library. The outcome of the feature is implemented by using the len function to compute the length of each tweet, and results represented in numeric data.

***Syntactic features*:** Syntactic feature performs a significant function in providing information regarding the tweets text syntactic structure. In this study, three sets of features that include POS feature, interjection word, & laughing expression are defined as syntactic features and are extracted from the processed tweet’s content. *To extract the syntactic feature*, this study employed NLTK tokenizer library to perform tokenization task on the processed tweets. Firstly, we extracted the POS feature using the parts of speech dictionary as the basis, and the count of its presence in the sarcastic text is taken. We only focused on the parts of speech details with some emotional contents such as nouns, adverbs, and adjectives. Furthermore, the mapping of each of the POS tags and each corresponding POS group was established, and only the tokenized words that correspond with the chosen three parts of speech groups as aforementioned were preserved in the text. The study employed the same framework used in [62] and extracted ADV+ADJ+N (adverb, adjective, and noun). Secondly, to extract the second feature, we identified laughter words that are used to express pleasures or joy. Thus, we added laughing features, which is the sum of internet laughs, represented with lol, hahaha, hehe, rofl, and imao, which we refer to as a new punctuation way. The feature is extracted by creating a dictionary list that contains the most common laughing words and using it to find the frequency of such words. Then, the frequency of such words present in the text was computed and added as a feature. The third feature is extracted by identifying interjection words such as woo, oh, wow, etc. in the tweets and the frequency of interjection words is computed and added as a feature.

***Emoticon feature*:** Emoticons are a pictorial representation of facial expression using punctuation and letters. A study on sarcasm analysis conducted in [63] noted that emoticons play a significant role in uttering sarcastic statements because it expresses the user’s mood. For instance, a smiley emoticon with negative situation words produces a sarcastic utterance and vice-versa. In this class of feature, emoticons that consist of positive emoticon like :-(, :(, :-|, ;-(, ;-<,|- {, negative emoticon like :-), :), :o, :-}, , ;-}, :->, ;-), and sarcastic emoticons such as (, [:, ;], -?[), p, P] are considered in this study. Emoticons are usually employed in ironic or sarcastic expressions. People use these emoticons to make a joke or funny in a situation when using sarcasm as a wit. To extract emoticon features, this research employed regular expressions to identify the use of emoticons that consists of Sad, Happy, Laughing, Surprise, and Winking by computing their frequency in each tweet. Then the frequencies obtained are regarded and added as a feature set.

***Lexical features*:** In this study, the Bag-of-Words model, which uses the term frequency inverse document frequency (TF-IDF) to represent a lexical feature was employed. Bag of Words based features is the most useful feature in the sentiment analysis studies. The lexical level feature uses TF-IDF to obtain the most descriptive terms in tweets data. To extract the lexical feature using the Bag-of-Words model, a pre-processed step is performed on the tweets dataset to eliminate the microblog typos and internet slangs. Next, a tokenizer, an NLTK library, is employed to tokenize the whole tweet dataset by splitting the tweets into individual words, also known as a token. Furthermore, a dictionary list is constructed based on the extracted words. Lastly, the TF-IDF feature is produced by employing the built-in function in Weka, which is then utilized as an input to the machine learning algorithm. Thus, the Bag-of-Words feature extraction process was performed in the Weka machine learning algorithm environment using the “StringToWordVector” function found in Weka.

***Hashtag features*:** Sometimes, emotional content is expressed by using hashtags. The hashtag is employed to disambiguate the actual intention of the Twitter user to pass a message. For instance, in a tweet, “Thanks a lot for always helping me, #i hate you.” In this utterance, the hashtag “#i hate you” shows that the user is not really expressing thanks to the intended but tremendously hating him for not helping him when the need arises. We call the above expression a negative hashtag tweet. Hashtag features could be a positive or negative hashtag. In this study, three sets of hashtag features are defined: a positive hashtag, a negative hashtag and the co-existence of the positive and negative hashtag. *The hashtag feature is extracted by creating a dictionary* that consists of a list of negative hashtag words such as “#hate, #pity, #waste, #discrimination, etc.”, and list of a positive hashtag such as “#happy, #perfect, #great, #goodness, etc.” However, using this dictionary, the number of positive hashtags and negative hashtags present in the tweet text is computed and added as a feature. The third feature is extracted by checking the co-existence of positive hashtags and negative hashtags in the same tweet. However, if there is co-existence in the same tweet, an integer one (1) is measured; otherwise, zero (0) is measured. Thus, the three sets of features are extracted and added as a feature set.

***Discourse markers*:** In social media platforms, people use various ‘discourse markers’ in making utterances. It has definite functions and aids in expressing an idea. Discourse markers such as temporal compression and counter-factuality have been utilized in irony detection study [64]. It is used to mark the upcoming words’ relationship to previous discourse (utterance used in a social context). This feature is very important in sarcasm identification because it helps comprehend utterances by providing a preview of what’s coming up. Counter-factuality concentrates on implicit marks, that is, discourse words that suggest contradiction or conflicts in a text. For example: yet, nevertheless, nonetheless, about, etc. On the other hand, temporal compression concentrates on identifying words associated with opposition in time, i.e. words that show a sudden change in description. Temporal compression can be represented using temporal verbs like suddenly, abruptly, etc. *A dictionary list that consists of a list of counter-factuality and temporal compression words is created to extract discourse marker features*. Using this dictionary, the number of counter-factuality and temporal compression words present in the tweets are computed and used as a feature for discourse marker.

***Semantic (word embedding) feature*:** Word embedding features employed to extract the semantic features are Global Vectors (GloVe). GloVe embedding is a very powerful word embedding learning scheme that learns vector representation of words employing dimensionality reduction on the co-occurrence matrix (a count-based model). This is done by constructing a large matrix of co-occurrence information, with the content of information on how frequent each “word” stored in rows appear in the column. It is a type of unsupervised technique used to obtain a meaningful vector that corresponds to individual words in a corpus [65]. In this model, different words repel against each other, where similar words cluster together. In GloVe, the counts’ matrix is pre-processed by normalizing the counts and log smoothing them. With GloVe embedding, one can use the co-occurrence matrix to obtain a semantic relationship between words [66]. One of the benefits of GloVe over other word-embedding schemes like word2vec is that GloVe does not capture only the local context information of the words (local statistics), but also captures word co-occurrence, also known as global statistics in a corpus to obtain word vectors. The GloVe allows parallel implementation, which makes it easy to train on a large corpus. It also combines the best features of two model families; the local content window methods and the global matrix factorization to create a new one [66].

- *Feature extraction and fusion* *process algorithm*

Definition of terms.

rt: raw tweet data
n: number of a row in the tweet
P: pre-process tweet data
℘t: Tokenization Function
℘c: Special Character removal function
℘l:: Lower Case conversion function
℘n: Number Value Removal function
℘w: Stop word removal function
℘s: Spell Checking function
ℑS: Sentiment related feature extraction function
ℑP: Pragmatic (punctuation) feature extraction function
ℑL: Lexical feature extraction function
ℑH: Hashtag feature extraction function
ℑDM: Discourse Markers feature extraction function
ℑG: Semantic (Glove embedding) feature extraction function
ℑE: Emoticon feature extraction function
ℑLM: Length of microblog feature extraction function
ℑST: Syntactic feature extraction function
IFV: Individual feature vector
φ: Feature fusion function
FFV: Multi-Feature fusion vector

### Algorithm 1: Data pre-processing, feature extraction and feature fusion process

**Input: Raw Twitter data** **(rt)**

**Output: Sets of features and feature fusion as input to machine learning classifiers**.

Procedure: FeatExtract (rt)

1: i ← 1
2: While i < = n
3: rt ← LOAD rt (i) from tweet data
4: rt_s ← ℘s (rt)      // perform spell check on the raw tweets
5: rt_w ← ℘w (rt_s)     // stop word removal from the raw tweets
6: rt_l ← ℘l (rt_w)      // convert raw tweet to lower case
7: rt_c ← ℘c (rt_l)      // remove special character from tweet
8: rt_n ← ℘n (rt_c)       // remove numerical values
9: rt_t ← ℘t (rt_n)          // tokenize the tweets
10: P(i) ← rt_t              // pre-process tweets
11: i ← i + 1
12: END
13: i ← 1
14: While i < = n
15: P ← LOAD P(i) from pre-processed tweet data
16: FS←ℑS(P(i))    // extract sentiment features from the pre-process tweet
17: FP ← ℑp((Pi))    // extract pragmatic features from the pre-process tweet
18: FL ← ℑL (P(i))   // extract lexical feature from the pre-process tweet
19: FH ← ℑH((Pi))   // extract hashtag feature from the pre-process tweet
20: FDM ← ℑDM((Pi))  // extract discourse markers feature from the pre-process tweet
21: FG ← ℑG(P(i))   // extract semantic features from the pre-process tweet
22: FE ← ℑE((Pi))   // extract emoticon feature from the pre-process tweet
23: FLM ← ℑLM((Pi)) // extract length of microblog feature from the pre-process tweet
24: FST ← ℑST (P(i))   // extract syntactic feature from the pre-process tweet
25: IF← [FS, FP, FL, FH, FDM, FG, FE, FLM, FST]   // sets of features extracted
26: WRITE IF      // append the extracted features to file
27: i ← i + 1
28: END
29: i ← 1
30: While i < = n
31: FF ← φ (FS, FP, FL, FH, FDM, FG, FE, FLM, FST)    // fusion of all feature sets
32: WRITE FF          //Append the feature vector
33: END

The steps of creating the feature vector for the proposed feature fusion can be explained as follows. The step is divided into three segments: data pre-processing, feature extraction, and feature fusion. In the data pre-processing, the raw tweet data *rt* is first loaded into memory. Next, six different pre-processing operations are performed before extracting discriminative features using the pre-processing (℘*)* function. It includes correction of a misspelt word using ℘_*s*_ on a raw tweet, stop word removal from the raw tweet by applying ℘_*w*_ on *rt*, lower case conversion of all words by applying ℘_*l*_ on *rt*, number value removal by applying ℘_*n*_ on *rt*, tokenization of sarcastic expression into a unique token by applying ℘_*t*_, lastly, the processed tweets *rt* is stored in storage location called *P*. In the feature extraction stage, on the other hand, the processed data is loaded to the memory for sarcasm classification. For every processed tweet, a set of the feature in a numerical form is extracted using the feature extraction function represented with *ℑ*. These features include Sentiment features (FS), Pragmatic features (FP), Lexical features (FL), Hashtag feature (FH), Discourse markers feature (FDM), Sematic (Glove Embedding) features (FG), Emoticon feature (FE), Length of microblog feature (FLM), and Syntactic feature (FST) features. Each feature is extracted in a numerical form, also referred to as an individual feature (IF). Furthermore, the feature fusion operation is performed using the fusion function represented with φ. The feature fusion involves fusing FS, FP, FL, FH, FDM, FG, FE, FLM, AND FST using φ to form the Multi-feature fusion (MFF). Finally, the fused feature is converted to the ARFF file format and provided as the input to classifiers for the classification step.

### 3.5 Classification models

This section discusses the machine learning algorithm, referred to as the classification model employed in this study. ML studies the algorithm that it can learn from and makes a prediction on data [67]. It is usually referred to as the model training stage that comes after the feature extraction stage. The extracted features from the dataset were used to construct a machine learning algorithm for sarcasm classification. That is, the machine learning algorithm has been utilized to classify tweets as either sarcastic or non-sarcastic. Numerous classifiers such as LR, DT, RF, KNN, and SVM, have been tested in different experiments to select the best classifier for sarcasm prediction. However, the decision on choosing the best classifier for a particular dataset is quite challenging. In the existing studies, two or more machine learning algorithms are tested to find the best algorithm since it is difficult to find a single classifier that can attain the best performance in all application domains [55]. This is because of the variations in the philosophy of the learning process. Thus, five different classifiers that include the Decision tree, Support Vector Machine, Logistic Regression, K-Nearest Neighbor, and Random Fores, has been employed to determine the model performance of the feature fusion framework for sarcasm identification. As a guide in selecting machine learning algorithms to be utilized in this study, three points have been employed to scale down the selection. First, specific literature on the classification algorithm for sarcasm detection is essential in the selection of specific classifiers. The distinction of the machine learning model may be restricted to a particular domain [56]. Therefore, the literature survey carried out in section 2 serves as a guide in selecting the classifier. Second, a text mining study review was also used to guide in model selection [57,58]. Third, the comparative results on comprehensive datasets are also guided in the selection of classification algorithms [59]. Thus, the machine learning algorithm such as SVM, KNN, RF, DT, and LR classifiers found in WEKA [68] has been tested in this multi-feature fusion framework.

The classifiers are briefly explained below.

**Decision tree (DT)**: DT is a learning algorithm that employs a tree structure model to decide [69]. It is a model that does not have a parameter but can easily handle the feature’s interaction. DT is based on the classification rule that displays a DT derived from a disorder class to an irregular instance [70]. It relies on feature value, for instance, classification using a sorting algorithm. The tree consists of paths, leaf, and decision nodes (a representation of each instance to be classified) and branch (a representation of the value that the node can undertake) [71]. The classification of instances starts from the root node and relies on its feature value for sorting. Overfitting is a major drawback in DT because of its ability to fit all the classes of data together with noise, which can affect its performance. To address the overfitting problem in DT, a multi-classifiers system like RF can be employed.

**Random forest (RF):** Ensemble classifiers have recently received increased attention due to their robustness and accuracy to noise when compared with single classifiers. Random forest is a powerful ensemble classifier of decision trees, which combine multiple decision trees. The notion of combining many classifiers grants the random forest special features that substantially differentiated it from the traditional tree classifiers. Like the single decision tree classifier with outliers or noise, which may affect the overall model performance, the RF classifier provides randomness to overcome such a problem due to its robustness to noise and outliers. The random forest does not only provide randomness to the data but also the features. Random forest makes use of similar concepts found in bootstrapping and bagging classifiers. This is carried out by increasing the diversity of trees, causing them to grow from different training data subsets created via bootstrap aggregation [72].

**Support vector machine (SVM):** SVM is a binary linear classifier proposed by Cortes and Vapnik [73]. A non-probabilistic supervised learning algorithm that uses high dimensional space to create a set of hyperplane for the separation of data into classes with the aid of training data [74]. However, the model is constructed using the training data to predict the target value, providing only attributes of test data [75]. SVM is a commonly used text classification algorithm requiring selecting the superlative hyperplane to classify the problem instances appropriately.

**Logistic regression (LR):** LR is a linear predictive model that classifies the probability of event occurrence as a linear function of a predictor variable class [76]. In the LR algorithm, the decision boundaries are usually made by employing a linear function of the features. Logistic regression aims to augment the probability function to recognize the document class label. The purpose of parameter selection in the LR is to attain the maximum conditional probability [77]. Despite LR’s promising result, the class variable usually generated is out of (0–1), which is not suitable for the probability range.

**K-nearest neighbor (KNN):** KNN is an instance-based machine learning model usually utilized for regression and classification tasks. In this form of a model, the identification of the class label for each instance depends on the k-nearest neighbor of that instance. Thus, the majority voting approach is employed in the neighbor instance to decide the class label. However, in this classification system, each neighbor’s majority vote is assigned to its class instance (i.e. the k-nearest neighbor’s most common class instance) [78].

Definition of terms

U:  U: Lexical feature content.
W:  W: Eight other groups of features
V:  V: Classification label.
C:  C: Lexical-based sarcasm tendency feature

### Algorithm 2: Two stages classification of the proposed framework

**Input:** Training set T = {(U1, W1, V1), (U2, W2, V2), … ….,(Un, Wn, Vn)}; 2 classifiers k1, and k2; a testing object M = (u, w);

**Output:** The label of M;

1: Train:
2: create lexical feature training set T1 = {(U1, V1), (U2, V2), …,(Un, Vn)};
3: train k1 on T1;
4: for i = 1to n do
5:   apply k1 on Ui to get C;
6: End for
7: create fusion feature training set T2 = {(C1, W1, V1), (C2, W2, V2), …, (Cn, Wn, Vn)};
8: train K2 on T2;
9: Test:
10: apply k1 over M = (U) to obtain its label CU;
11: apply k2 over M1 = (CU, w) to obtain its label V;
12: Return V;

To the best of our knowledge, this is the first study on sarcasm identification that proposes a novel feature extraction algorithm and two stages classification algorithm for sarcasm identification by considering the lexical feature in the first stage and fused features in the second stage. The impact of ‘Algorithm 1 and Algorithm 2’ are as follows

- The feature extraction algorithm provides the stepwise representation for extracting the proposed multi-feature in the form of a feature vector, which is employed as an input to the machine learning algorithm.
- The pre-processing segments of the feature extraction algorithm help in the data preparation stage, such as tokenization, POS tagging, and data normalization, such as stemming and lemmatization, before the actual feature extraction process.
- Both algorithms do not depend on any programming language; thus anyone without programming knowledge can easily understand it.
- Th two-stages classification algorithm helps describe the stepwise procedure for classifying tweets into sarcastic or non-sarcastic to obtain the predictive performance, which makes it simple to comprehend.

### 3.6 Feature analysis and selection

Analysis of features is conducted to identify the most performing features. This feature selection technique is essential in any text classification tasks. It can reduce the computation time and eliminate the irrelevant features that do not contribute to the classification performance. As a result, some features are insignificant and do not add any value to the classifier’s performance; hence, two feature selection algorithms have been investigated, namely Pearson correlation and information gain, to find the discriminative power in each feature [79]. Hence, we employed the selection technique in the second stage of classification to test if the results can be enhanced

## 4. Experimental settings

The classification experiment was carried out to analyze the sarcasm expression (sarcastic and non-sarcastic) in a given tweet. All experiments were performed on a system running on window 10 with 64-bit operating systems. The system uses an Intel Core™ i7-4770 CPU @ 3.400GHz with 16GB Random Access Memory (RAM) capacity. The pre-processing and feature extraction task was performed in the Jupyter notebook environment, a python programming language development environment. Subsets of features explained in section 4.1 have been employed in the sarcasm analysis experiment as input to various machine learning algorithms. We have experimented with five different machine learning models that consist of LR, DT, RF, KNN, and SVM to estimate sarcastic sentiment in the given tweets. The purpose of employing different models is to get the best performance result. This study utilized a 10-folds cross-validation approach in performing all experiments [80]. The machine toolkit WEKA 3.9, consisting of various machine learning algorithms executed in Java, was used as the experimental environment to perform classification and feature selection experiments. The default settings were used during the experiment. Four standard evaluation metrics such as precision, recall, f-measure, and accuracy were tested and weighted over both classes during the experiments. The list of the experimental environment settings is depicted in Table 5.

**Table 5:** Lists of the experimental environment.

S/N	Experiments	Environment
1	Data-preprocessing and normalization	Python programming environment
2	Feature extraction and feature fusion	Python programming environment
3	Feature selection	Weka tool kit environment
4	Sarcasm classification	Weka tool kit environment

Moreover, this section presents the parameter settings of classifiers used during the experiment. The same set of parameters were employed for all the experimental settings to measure the multi-feature fusion framework for sarcasm identification. The classifiers parameter turning are depicted in Table 6.

**Table 6:** Parameter optimization and tuning values of classifiers.

Classifier	Parameters	Values
Support Vector Machine	Batch size	100
	Kernel	Polykernel-E1.0-C250007
	Complexity	1.0
	Epsilon	1.0E-12
	Tolerance parameter	0.001
Logistic Regression	Batch size	100
	Ridge	1.0E-8
	Maxlts	-1
K- Nearest Neighbors	Batch size	100
	K	10
Decision Tree (J48)	Batch size	100
	Confidence factor	0.25
	Number of fold	3.0
	MinNumObj	2.0
	Seed	1.0
Random Forest	Batch size	100
	numExecutionSlots	1.0
	numIterations	100
	Seed	1.0

### 4.1 Evaluation measures

Evaluation measures are the performance indicators that have been established to measure the output of the experiment. Various evaluation matrices have been employed to measure the output of the classification algorithm. This research utilized ‘Precision’ as the major performance evaluation. However, other performance metrics such as recall, accuracy, and f-measure have been employed as a supplemental to evaluate the framework’s performance. Each classifier proves its sarcasm identification potential when evaluated by these metrics.

**Classification accuracy (ACC)** denotes the entire correctness of the classification result. It measures the fraction of true positive and true negative attained by the classified instances over the whole instances, represented in Eq 1. TP, TN, FN, and FP represent the true positive number, true negative number, false-negative number, and false-positive number.

**Recall (REC)** measures the fraction of true positives over the summation of a true positive and false negative. In other words, it computes the sum of the tweets correctly classified as sarcastic over the sum of sarcastic tweets. It is depicted in Eq 2

**Precision (PRE)** is a measure of the fraction of TP over the TP and FP. It determines the number of tweets that have been accurately classified as sarcastic over the whole tweets that were classified as sarcastic. It is indicated in Eq 3.

**F-measure (F-M)** is a performance evaluation that merges precision and recall by computing their harmonic mean. It has been previously employed in the classification study as the overall measurement of classifiers’ performance as it considers precision and recall [81]. F-M assumes the values of 0 and 1. It is represented in Eq 4.

**Confusion matrix (CM):** CM, also known as the error matrix, is a unique table representation that gives the picture of the classifier’s execution, especially the supervised learning classification. The confusion matrix consists of two instances (“predicted” and “actual”) of the same sets of classes. The negative is discarded, whereas the positive is identified. Thus, after the classification, true positive is the instance that is accurately classified, whereas false positive is not correctly classified. The false-positive instance symbolizes a type 1 error, indicating that the number of instances is not correctly indicated as positive. On the other hand, the true-negative are those instances that are correctly discarded, and false negatives denoted the incorrectly classified instance. False-negative symbolizes type 2 error, indicating that the number of instances is incorrectly classified as negative. The pictorial diagram of the confusion matrix is depicted in Table 7.


$$Acc=\frac{TP+TN}{TP+TN+FP+FN}$$ (1)

$$PRE=\frac{TP}{TP+FP}$$ (2)

$$REC=\frac{TP}{TP+FN}$$ (3)

$$FM=2*\frac{PRE*REC}{PRE+REC}$$ (4)

**Table 7:** Confusion matrix.

	*True condition*	
***Predicted condition***	***TP***	***FP (type 1 error)***
	***FN (type 2 error)***	***TN***

## 5. Empirical results and discussions

This section provides and discusses the predictive results of the experiments on the Twitter dataset. All five classifiers were run on the proposed multi-feature fusion using 10-folds cross-validation. Precision is used as a major evaluation. However, we also reported the performance of the f-measure, recall, accuracy on each classifier. The experimental results were generally very suitable and assisted in predicting the best classifiers for sarcastic classification. The results are presented in two segments.

### 5.1 Results based on lexical feature only

**Fig 3:**
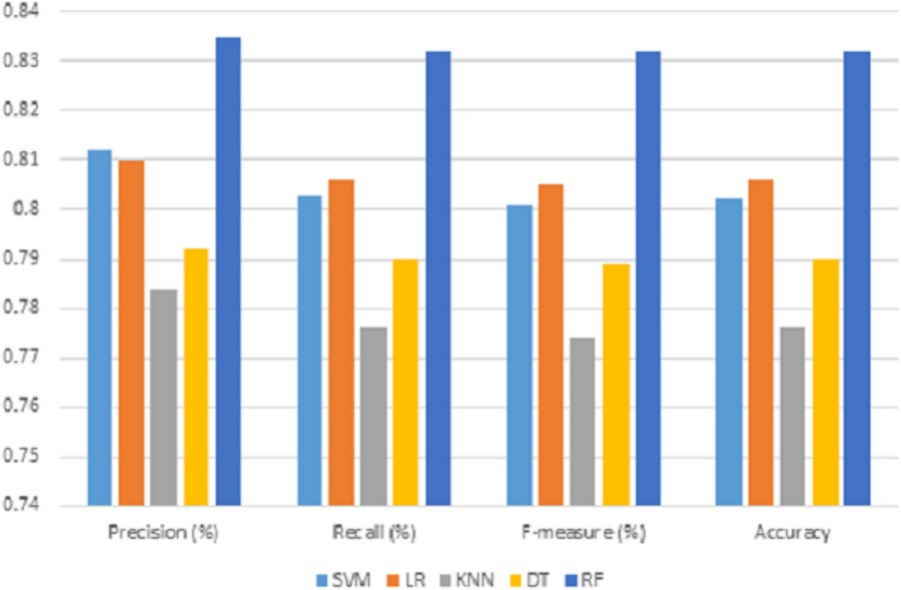
Performance results of different classification algorithms on the lexical feature only.

The predictive performance result based lexical feature is presented in Table 8 and the visualization of the results is depicted in Fig 3. It can be observed from Table 8 that the performance results of precision, recall, f-measure and accuracy falls in the range of 78% and 83.5%. The values show that all the classifiers understand the sarcastic expression based on all the features. The results show that the random forest classifier attained the highest performance precision, with 83.5% over all the classifiers. The results also show that it outperformed other classifiers in terms of f-measure, recall, and accuracy. Even LR also showed good performance in the classification, indicating that it understood the sarcastic expressions.

**Table 8:** Performance results obtained by considering lexical feature only.

Classifiers	Precision (%)	Recall (%)	F-measure (%)	Accuracy (%)
SVM	0.812	0.803	0.801	0.802
LR	0.810	0.806	0.805	0.806
KNN	0.784	0.776	0.774	0.776
DT	0.792	0.790	0.789	0.790
RF	0.835	0.832	0.832	0.832

Moreover, Table 8 also shows that low-performance results were recorded in KNN and DT classifiers, whereby the two classifiers obtained precision results of 78.4% and 79.2%. Having the least performance does not mean that both classifiers do not understand the sarcastic utterances but still can attain better performance. It shows that both classifiers had a low understanding of sarcastic expression based on the lexical features. The SVM and LR classifiers show a negligible difference in precision performance. A conclusion can be made based on the result that the performance of the RF is attributed to the ensemble properties.

In Fig 3, the precision-based analysis of the classifiers on cross-validation with 10-fold cross-validation shows the dominance of random forest classifier. The random forest classifier-based model attained the highest precision (83.5%), f-measure (83.2%), recall (83.2%), and overall accuracy (83.2%) in comparison with second-highest precision (83.5%), in the case of support vector classifier. On the contrary, the logistic regression classifier attained the second-highest recall (80.6%), f-measure (80.5%), and overall accuracy (80.6%) when compared with other classifiers. Lexical features are content-based features, which has the drawback of loss of contextual information. To address the issue, some contextual features are fused with the lexical feature for classification.

### 5.2 Results based on the fused features

**Fig 4:**
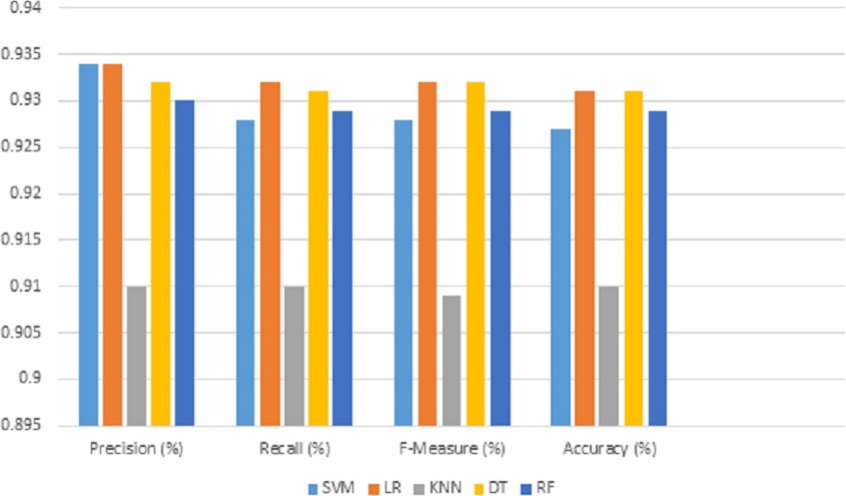
Performance results of different classification algorithms on the fused features.

The predictive results based on the fused features is presented in Table 9, and the visualization of the results is depicted in Fig 4. As shown in Table 9, the proposed Framework (Multi-Feature Fusion) was effective in the model evaluation. The table shows the simulated result by comparing different classifiers on sarcasm analysis, whereas Fig 4 shows the results’ visualization. It can be observed from the table that the DT and RF had a good classification performance. It shows that both classifiers understood the sarcastic utterances, which further shows that both classifiers can effectively classify sarcastic utterances. We can also imagine from the table that the DT classifier outperformed KNN. It can also be observed that the last result is obtained with the KNN classifier, yet it still can produce good results. It is obvious from the experimental results that out of the five models tested, LR and SVM are competing in terms of precision by attaining 93.4% precision. However, the LR outperforms the SVM in terms of f-measure, recall, and accuracy.

**Table 9:** Performance results obtained by considering fused features.

Classifiers	Precision (%)	Recall (%)	F-Measure (%)	Accuracy (%)
SVM	0.934	0.928	0.928	0.927
LR	0.934	0.932	0.932	0.931
KNN	0.910	0.910	0.909	0.910
DT	0.932	0.931	0.932	0.931
RF	0.930	0.929	0.929	0.929

Table 8 presented the experimental results by considering the lexical features only. Lexical features are content-based features, which has the drawback of loss of contextual information. However, the highest predictive result obtained by considering the lexical features is 83.5% precision on the RF classifier. However, when contextual features were added to the content-based feature (Lexical), a significant improvement in predictive performance results was obtained (0.934 precision), as depicted in Table 9. It can be seen from Table 9 that RF attained a precision of 0.930, SVM attained a precision of 0.935. LR attained a precision of 0.935, and KNN attained a precision of 0.910, and DT attained a precision of 0.932, which shows the significance of contextual features in sarcasm classification.

Moreover, when the results are compared with the results obtained with the lexical feature (BoW), it can be observed that there is a significant improvement in the performance in all the models. For instance, the RF classifier attained more than 9.5% and 9.7% for precision and f-measure, respectively, which shows the significance of the proposed multi-feature fusion framework in the sarcasm analysis task.

Thus, the results of the experiments show that the proposed multi-feature fusion framework in the sarcasm analysis task that consists of lexica, length of microblog, hashtag, discourse markers, emoticons, syntactic, pragmatic, semantic (GloVe embedding), and sentiment related features enhanced the predictive performance of the sarcasm classification.

### 5.3 Results obtained by classifiers with feature selection

We experimented with all the five classifiers on the proposed multi-feature fusion framework to recognize the most discriminative features that may enhance the classifiers’ performance and lower the classification time. Thus, Pearson correlation and information gain feature selection algorithm were investigated in this analysis. All the feature sets of Multi-feature fusion were tested, and features with discriminative power that could enhance the predictive performance were selected. Tables 10 and 11 compare the results of the five classifiers with each feature selection algorithm. The comparison of Table 9 with Table 10 indicates that the use of the Pearson correlation feature selection algorithm on the proposed Multi-feature fusion has slightly enhanced the precision performance for RF (0.947), KNN (0.917), LR (0.940), SVM (0.937) and DT (0.935). Table 11 depicts the experimental results attained by employing the information gain feature selection method on the proposed Multi-feature fusion. The comparative results with Table 9 show a slight improvement in precision with RF (0.944), KNN (0.917), (0.936), SVM (0.937), and LR (0.940) remained the same.

**Table 10:** Performance results attained on fused features using Pearson correlation.

Classifiers	Precision (%)	Recall (%)	F-measure (%)	Accuracy (%)
SVM	0.937	0.933	0.932	0.933
LR	0.940	0.938	0.938	0.938
KNN	0.917	0.917	0.917	0.916
DT	0.935	0.934	0.934	0.934
RF	0.947	0.946	0.946	0.945

**Table 11:** Performance results attained on fused features using information gain.

Classifiers	Precision (s%)	Recall (%)	F-Measure (%)	Accuracy (%)
SVM	0.937	0.933	0.932	0.932
LR	0.940	0.938	0.938	0.937
KNN	0.917	0.917	0.917	0.916
DT	0.936	0.935	0.934	0.935
RF	0.944	0.943	0.943	0.943

A classification algorithm constructed with feature fusion and feature selection is suggested to evaluate the predictive performance in terms of precision, f-measure, recall, and accuracy. Applying feature selection techniques shows that the two feature selection techniques (Pearson correlation and information gain) tested attained almost the same results except in RF classifiers. However, using Pearson correlation feature selection outperformed the information gain by attaining 94.7% precision over 94.4%, as shown in Tables 10 and 11, respectively. When the results obtained in Tables 10 and 11 are compared with the results obtained from Table 9, an increase in predictive performance is observed. Hence, there is a significant enhancement in classifiers performance in applying feature selection techniques. Thus, the reduction in the results in Table 9, compared with Tables 10 and 11, can be attributed to the presence of the null features (data sparsity) in the training sets. Consequently, applying the feature selection techniques can eliminate null features and enhances the predictive performance in sarcasm classification.

In the overall performance, it can be observed that RF outperformed all the four other classifiers by attaining a precision of 94.7%. We assumed that the random forest’s performance result of random forest is attributed to the ensemble scheme, whereby approximately 300 decision trees are combined and together with ten features to attain a consensus of sarcasm classification. RF classifier is one of the powerful learning models that train on various datasets, including large datasets, and it can handle large input features while parameters remain the same. The model approximates missing data due to its ability to maintain accuracy when there is missing data. It balances errors in the dataset even when there is an imbalance in class distribution.

### 5.4 Significance of the proposed multi-feature fusion

**Fig 5:**
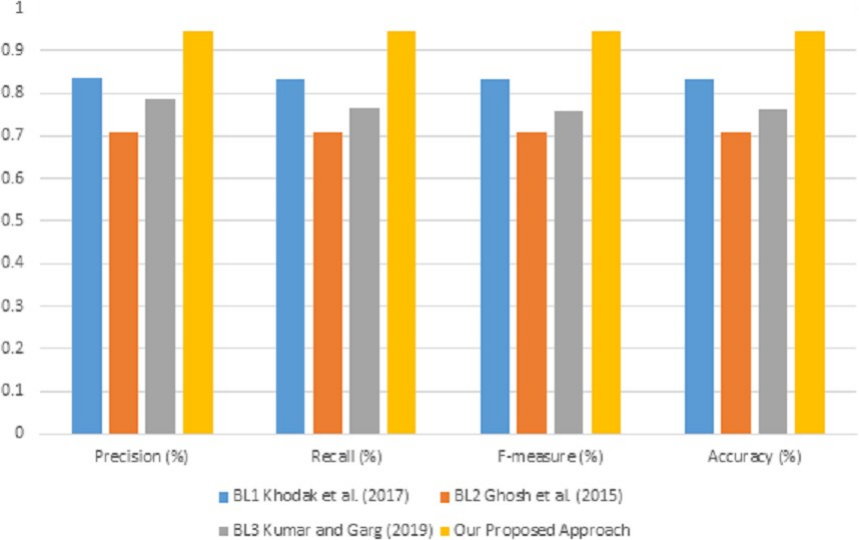
Results comparison of our proposed framework with the baseline approaches.

To measure the significance of the proposed framework, an extensive set of experiment was performed on our dataset to evaluate classifiers’ performance using four baseline approaches for sarcasm identification in Twitter data. The four baseline methods were established to compare with the proposed framework. The first baseline approach is based on the Bag-of-words (BoW) technique [22,23,35]. The second baseline is based on word embedding (word vector), which is another important baseline that uses a contextual word vector that includes GloVe [66] trained 42B corpus. Few studies have utilized GloVe for sarcasm detection tasks [82–84]. The third baseline is the feature fusion method proposed in [85], which utilized the fusion of pragmatic feature, sentiment feature, and Top-200 TF-IDF features to build the context using shallow classifiers. The fourth baseline is a proposed approach studied in [86] that proposed stacking ensemble feature-based sarcasm detection in Twitter. During the evaluation experiment, the first baseline attained a promising result with a DT classifier by obtaining a precision of 0.837. In baseline 2, a random forest classifier attained the best result by obtaining a precision of 0.710. Baseline 3 achieved the highest result with a RF classifier by obtaining a precision of 0.787, whereas baseline 4 obtained a highest precision results of 0.666 with a RF classifier. We compared the best result attained from our proposed framework with those obtained from three baseline approaches. The comparison results are depicted in Table 12. The last row of the table shows the performance of our proposed framework. With the random forest classifier, the best precision of 94.7% was obtained in our proposed framework using the Pearson correlation feature selection algorithm, which indicates the significance of the proposed multi-feature fusion framework for classifying tweets as sarcastic and non-sarcastic. Thus, our proposed framework outperformed baseline one by 11.2%, baseline two by 23.7%, baseline three by 16% precision, and baseline four by 28.1% during the cross-validation. In addition, our method also shows a relatively higher f-measure when compared with the baselines. In Fig 5, the visualization of the comparison is represented.

**Table 12:** Comparison of the proposed framework with baselines.

Baselines/Proposed Approach	Precision (%)	Recall (%)	F-measure (%)	Accuracy (%)
BL1 Khodak et al. (2017) [22]	0.835	0.832	0.832	0.832
BL2 Ghosh et al. (2015) [82]	0.710	0.709	0.709	0.710
BL3 Kumar and Garg (2019) [85]	0.787	0.764	0.759	0.763
BL4 [86]	0.666	0.610	0.574	0.610
Our Proposed Approach	0.947	0.946	0.946	0.945

#### 5.4.1. Discussions

The first baseline uses bag-of-words feature representation. However, simple representation features using bag-of-words, in which each word in the dataset is regarded as a feature, may lead to inadequate features for constructing the machine learning model. This phenomenon is noticed in the predictive performance results in which the proposed framework performed better than bag-of-words. This is due to the drawback imposed in the BoW feature engineering technique loss of contextual information and training data sparsity problem. Thus, results obtained using the BoW feature engineering approach are not comprehensive and generalized for sarcasm identification.

The second baseline uses word embedding (word vector) feature representation to classify a tweet as sarcastic or non-sarcastic. Low-performance results were also recorded on the second baseline method. The brain behind the low performance on using the word embedding feature is due to the limitation inherent in such representation. One of the major limitations of word embedding is that it ignores the sentiment polarity of words [87,88]. Though word embedding based word vector captures the word’s context but ignores the sentiment polarity of words in the expression. Thus, the word embedding (word vector) feature lacks enough sentiment information in performing sarcasm classification, and it does not precisely capture the overall sentiment of the sarcastic expression.

The third baseline is based on combining various features that included utilized pragmatic feature, sentiment feature, and Top-200 TF-IDF features to build the sarcasm classification using shallow classifiers. Low-performance results are also observed in the baseline because the approach did not utilize word embedding-based features even though it included the sentiment-related features. Word embedding is imperative in sentiment analysis study, especially in the sarcasm classification task, as it captures the word semantics in the sentence. Due to the deficiency of word embedding-based features, the word co-occurrence in the text is not captured. Thus, more features that include word embedding should be explored for effective classification and performance enhancement.

The fourth baseline is based on the combination of various features such as lexical, emoticon, internet slang, and hyperbolic feature. This baseline attained the lowest performance. Though this baseline contains some discriminative features for sarcasm classification yet, these features are not adequate and comprehensive enough because the pragmatic feature is missing. Pragmatic features are markers that describe the “meaning in the context.” Also, some important features that are paramount for sarcasm classification, such as sentiment-related features and context embedding features, are missing in the study. As a result, low-performance results were obtained on the baseline 4 study approach

Therefore, the comparison results indicate that the multi-feature fusion framework utilizing the proposed features is more effective for sarcasm classification when compared with the four baseline approaches.

## 6. Conclusion

Sarcasm identification has been a crucial challenge in the NLP study. The sarcasm identification task is a classification problem aimed at distinguishing sarcastic utterances from the non-sarcastic counterpart. Accurate identification of sarcasm can enhance the sentiment classification and opinion mining study. In this study, we have considered a multi-feature fusion framework for sarcasm classification using machine learning approaches to overcome the aforementioned limitations in the most related techniques by addressing the context of words and data sparsity in sarcastic expression using two classification stages by employing various classifiers (SVM, DT, KNN, LR, and RF) to build machine learning algorithm for sarcasm prediction. We conducted extensive experiments to measure the performance of the five selected classifiers. Two feature selection algorithms were investigated: Information gain and Pearson correlation to identify the most discriminative features. We experimented with various feature selection techniques to select the features with the substantial discriminative ability to enhance the predictive performance results. Precision was utilized as the major performance measure due to its robustness in measuring classifiers. The highest classification was attained by employing classifiers that use feature selection technique to select the features with discriminative power. Random forest classifier with Pearson correlation feature selection technique attained the highest precision (0.947) and f-measure (0.946). Comparing our highest results with baselines shows the importance of the proposed framework for sarcasm identification. Thus, the results show that bolstering lexical features with some contextual features such as semantic features, discourse markers, NLP feature (POS), sentiment, pragmatics, and hashtag features, resolves the problem of the context of words in sarcasm expression, whereas performing feature selection on fused features to select the discriminative features eliminates null features, thereby reducing data sparsity.

Despite the promising result obtained on the proposed feature fusion framework, few limitations are identified in the approach. One, the feature extraction approach was made manually/ handcrafted. However, extracting features via this approach for sarcasm detection is Labour intensive. Thus, an ample amount of time is needed for the feature extraction stage. Besides, the feature extraction approach as mentioned above is application-dependent and cannot be generalized to a new domain. Secondly, the proposed multi-feature fusion framework is developed with the only Twitter dataset. To ensure the comprehensives in this framework, other social media corpus such as product reviews, internet argumentation corpus, and Reddit news, are required for sarcasm identification study. Thirdly, the constructed machine learning algorithm can only classify sarcastic text composed in the English language. However, the proposed framework can also be employed for sarcasm classification in other languages if the extracted features for model training are represented in such languages

In the future, the proposed framework can be employed to enhance sentiment classification and opinion mining due to its ability to recognize sarcastic utterances in Twitter data. The scope of this work can also be extended to investigate the transfer learning approach for sarcasm identification using the bidirectional encoder representation for transformer (BERT) model. Accordingly, the advancement in using text-image, in which text is represented as an image that also shows the effectiveness of social media utterances, is another promising research direction for sarcasm classification. Also, we are currently conducting different deep learning-based approaches for sarcasm detection and hope to publish our findings soon.
